# The Melbourne Hospitai

**Published:** 1907-10-19

**Authors:** 


### THE MELBOURNE HOSPITAL.

### THE ELECTION OF THE HONORARY MEDICAL OFFICERS

The Melbourne Hospital was founded in 1846, and is supported by voluntary contributions, aided by a Government grant of £10,000 a year. It is the clinical hospital for the medical school of the University of Melbourne, and may therefore be regarded as the most important hospital in Victoria.

It is the practice at this institution to elect the honorary medical officers every four years. This plan has given rise to abuses which the Governors should make it their business to prevent for the future by amending their by-laws and regulations. We fully described the methods in a leading article in our issue of August 31, and need only repeat here that we most strongly deprecate a system of election which puts the responsible choice of hospital physicians and surgeons to the tumult of a popular election in a manufactured and irresponsible constituency.

The constituency is manufactured and irresponsible from the circumstance that every person who pays £1 to the funds of the hospital becomes a governor for the year within which such payment is made. The *Melbourne Age* dated August 28 thus describes the election:—"The approaches to the Athenæum, where the elections were held, were busy all day, but after noon they were thronged to such an extent that voters found it difficult to get through. Mr. Robert Showers, the returning officer, had a large staff of assistants at work, and the voting went off smoothly, but at one time the returning officer feared that the available supply of ballot papers would prove insufficient, and sent away for more. One distinctive feature of the election was the free use made of the 'faggot vote.' At the last election this method of 'working' the poll was not permitted, but the returning officer was on that occasion threatened with a writ for refusing votes so obtained and tendered. Mr. (now Mr. Justice) Cussons gave a legal opinion to the effect that any person was qualified to vote as soon as he had put his money entitling him to enrolment as a subscriber, and thus the Melbourne Hospital Committee, he advised, had exceeded their powers in prohibiting subscribers from voting till they had been registered on the rolls for three months."

At this election a small office was opened for the sale of votes, and upwards of 250 people who paid £1 each were registered as subscribers, and furnished with voting papers. Anything better calculated to injure the reputation of a great hospital has probably never before formed a feature in the election of members of the honorary medical staff.

The election partook of the nature of a Parliamentary contest. Considerable feeling was evinced in the poll, and a vigorous canvass was instituted on behalf of the candidates. Motor cars and carriages were employed in large numbers to bring voters to the poll. The number of votes polled was very large, the most popular candidates receiving upwards of 4,000 votes each. It is satisfactory to be able to report that in the result the Governors set their faces like a rock against the introduction of outsiders, by re-electing without exception the whole of the physicians and surgeons, as well as of the physicians and surgeons to out-patients. This result, as the *Age* well says, must be regarded as a decided expression of the opinion of the subscribers, "that the interests of hospitals and the public, for whose benefit they are established, are best served by keeping in their places of skilled responsibility professional gentlemen who have proved their fitness for their position by the work they have accomplished, and whose knowledge is augmented with the increase of experience thus gained as honorary medical officers to the Melbourne Hospital."

We venture to hope that this is the last occasion upon which the subscribers will ever permit "faggot-votes" to be used in an election for the office of honorary physician or honorary surgeon. The demands upon the purses of the individual members of the medical profession who serve hospitals in an honorary capacity are already large enough, and any hospital which sanctions the continuance of a system which renders it necessary for each honorary medical officer, to retain his position, to enter into a prolonged and expensive contest every four years is self-condemned as one where the administration fails to be efficient and up to date. The *Melbourne Age* is to be congratulated upon the stand it has made against the abuses of a system which has too long continued, and which we hope, with its powerful influence, may soon be relegated to the limbo of forgotten things.

